# Fatty Acid Composition and Antioxidant Activity of Milk from the Bulgarian Local Donkey Breed

**DOI:** 10.3390/foods15040614

**Published:** 2026-02-08

**Authors:** Nikolina Naydenova, Petya Veleva, Ana Georgieva, Kamelia Petkova-Parlapanska, Ekaterina Georgieva, Galina Nikolova, Yanka Karamalakova

**Affiliations:** 1 Dairy Science Department, Agricultural Faculty, Trakia University, Student Town, 6000 Stara Zagora, Bulgaria; 2 Department of Agricultural Engineering Agricultural Faculty, Trakia University, Student Town, 6000 Stara Zagora, Bulgaria; petya.veleva@trakia-uni.bg; 3 Chemistry and Biochemistry Department, Medical Faculty, Trakia University, 11 Armeiska Str., 6000 Stara Zagora, Bulgaria; kamelia.parlapanska@trakia-uni.bg (K.P.-P.); ekaterina.georgieva@trakia-uni.bg (E.G.); galina.nikolova@trakia-uni.bg (G.N.)

**Keywords:** donkey milk, fatty acid composition, antioxidant properties

## Abstract

Donkey milk has been increasingly studied in recent years and has been proposed to be a functional food. However, its components undergo changes during lactation, including its lipid profile and redox-related properties. This study analyzed the fatty acid composition, antioxidant parameters, and redox-modulating properties of donkey milk from the Bulgarian local donkey breed at three lactation stages (0–30, 31–60, and 61–90 days postpartum). Milk samples from 40 clinically healthy donkeys were grouped by days postpartum. A cross-sectional design with three lactation stage groups was used; one-way ANOVA tested group differences with Tukey’s post hoc test, and associations with days postpartum were evaluated using regression models. Fatty acid methyl esters were analyzed by GC-FID, and the atherogenic (AI) and thrombogenic (TI) indices were calculated. Antioxidant enzymes (SOD, CAT, and GPx-1), GSH, MDA, TAC, and EPR-based redox markers (DPPH, Asc•, ROS, NO•, TEMPOL, and 5-MSL) were analyzed. During lactation, monounsaturated fatty acids decreased (approximately 32% in the first month to ~30% by the third month), while AI increased from ~1.9 to ~2.2, and TI increased to ~2.5. SOD and GPx-1 activities increased with advancing lactation, while total antioxidant capacity decreased (213.4 to 199.7 µmol). DPPH radical scavenging activity remained stable during lactation. EPR-detected ROS and NO• values increased with advancing lactation stage, while thiol-bound 5-MSL decreased, suggesting a shift in the balance between oxidative challenge and antioxidant defense during lactation. Regression modeling confirmed a significant effect of lactation period on multiple compositional and redox-related parameters. Therefore, the stage of lactation should be taken into account when interpreting the biological value, redox stability, and potential functional properties of milk, as well as when developing milk management and yield strategies.

## 1. Introduction

Donkey milk (DM) has a long history as both a nutritional source and a therapeutic agent. Contemporary research has increasingly focused on characterizing its unique biochemical constituents, revealing its multifaceted health benefits, and supporting gut homeostasis and redox balance [1]. In recent years, growing evidence suggests that donkey milk is compositionally closer to human milk (HM), positioning it as a potential alternative for infant nutrition, particularly in cases of cow’s milk protein allergy (CMPA) [2,3,4,5].

Cow’s milk protein allergy is mainly driven by immune reactions to bovine caseins (notably α_s1_-casein) and whey proteins such as β-lactoglobulin. Donkey milk differs from cow’s milk by having a lower total protein content, a higher whey-to-casein ratio, and a casein profile closer to human milk, which can reduce IgE cross-reactivity in many individuals with CMPA. Nevertheless, donkey milk is not universally hypoallergenic and occasional reactions have been reported; therefore, its use for CMPA should be evaluated on a case-by-case basis and under medical supervision [3,4,6].

DM has a characteristically low fat content, ranging from 0.1% to 3.8%, which distinguishes it from bovine milk. Its lipid profile demonstrates several nutritionally advantageous features: elevated concentrations of α-linolenic acid (C18:3n-3), higher proportions of total omega-3 fatty acids, reduced levels of saturated fatty acids, and a more balanced omega-6-to-omega-3 ratio compared to conventional cow’s milk [7].

Beyond its nutritional profile, donkey milk contains bioactive proteins with potential roles in redox homeostasis. Lactoferrin may limit metal-catalyzed oxidation through iron chelation and has been described as a modulator of redox- and inflammation-related mechanisms in recent reviews [8,9]. Lysozyme, together with lactoferrin, also contributes to antimicrobial defense and may reduce inflammatory stimuli (e.g., microbial products) that promote reactive oxygen and nitrogen species generation, supporting a functional link between antimicrobial action and oxidative stress control [5,10,11].

A higher antioxidant capacity may improve oxidative stability of donkey milk and may contribute to dietary antioxidant intake, potentially supporting protection against oxidative-stress-related disorders [12].

Previous studies have compared donkey and cow milk and reported higher HO• radical-scavenging capacity for donkey milk, and found that DM has a higher HO• radical-scavenging ability (>1487.835 U/mL) compared to CM (1054.980 U/mL) [13]. On the other hand, during lactation, the rapid development of the mammary glands and the synthesis of large amounts of milk components increase energy requirements and metabolic rates. The increase in oxidative stress may raise the risk of limiting lactation potential [14].

Simos et al. [15] determined the antioxidant activity of DM by measuring its oxygen radical capacity (ORAC) and concluded that caseins and hydrophilic antioxidants such as vitamin C, vitamins E, and uric acid are the main factors contributing to this activity. Because mammary metabolism and milk synthesis change rapidly after parturition, both lipid composition and antioxidant-related components can vary across lactation. Recent stage-resolved studies using lipidomics have documented significant shifts in donkey milk lipid profiles across lactation, reinforcing the need for stage-specific evaluation of fatty acid quality indices and related functional claims [16,17]. Moreover, recent work emphasizes compositional evolution across lactation in donkey milk in the context of nutritional and functional food value, further supporting stage-specific profiling for correct interpretation of oxidative stress-related outcomes [11,18].

In recent years, consumer interest in donkey milk has increased in Bulgaria; however, data on the biochemical composition and health-related redox markers of milk from the Bulgarian native donkey breed remain scarce—particularly for integrative profiling that combines fatty acid quality indices with EPR-based redox readouts during early lactation. The novelty of this study is that it (i) provides breed- and region-specific fatty-acid profiling for Bulgarian local donkeys, (ii) reports lactation-stage dynamics of AI and TI together with a conventional antioxidant panel (SOD, CAT, GPx-1, GSH, MDA, and TAC), and (iii) integrates EPR-derived markers (ROS- and NO•-related signals, thiol/protein oxidation via 5-MSL, and TEMPOL conversion) to characterize redox-related changes during 0–90 days postpartum.

We hypothesized that (H1) fatty acid quality indices (AI, TI) and selected fatty acid classes change with lactation stage, and (H2) EPR-derived redox markers exhibit stage-dependent shifts consistent with altered oxidative challenge in later lactation.

## 2. Materials and Methods

### 2.1. Ethics Statement

This study was conducted at the Milk and Dairy Products Laboratory and the Laboratory of Biochemistry, Faculty of Medicine, Trakia University (Stara Zagora, Bulgaria). All procedures were performed under the institutional guidelines for animal research (Trakia University, Stara Zagora, Bulgaria). Ethical approval was not required under the institutional policy because the study involved non-invasive milk sampling during routine milking, and no experimental procedures were performed on the animals.

### 2.2. Animals, Feeding Management, and Milk Sampling

A total of 40 healthy lactating Bulgarian native breed donkeys were selected from semi-extensive conditions in the Sliven district, Seliminovo village, at the foot of the Stara Planina, Bulgaria. The animals were fed mainly on natural pastures with added grain feed (2.0–2.5 kg barley, twice daily), and water was provided ad libitum (i.e., free access at all times). Throughout the lactation period, the foals were kept with their mothers and separated only before milking.

Individual milk samples were collected during the first three months of lactation from animals aged 5–12 years and weighing 250–300 kg, all in good physical condition. Donkeys were milked manually twice daily during spring/summer (April–July 2022). Immediately after collection, milk samples were placed in a mobile refrigerator at 4 °C and analyzed without delay. The samples were randomly allocated into three independent groups according to lactation stage: up to 30 days postpartum, from 31 to 60 days postpartum, and from 61 to 90 days postpartum (*n* = 12, 16, and 12, respectively). Each animal contributed one sample to one lactation stage group; therefore, the dataset represents a cross-sectional design rather than repeated measurements.

### 2.3. Lipid Extraction and Fatty Acid Analysis

The extraction of DM fat was performed by the method of Rose–Gottlieb using diethyl ether and petroleum ether (Methodenbuch, Bd. VI, VDLUFA-Verlag, Darmstadt, Germany, 1985). Thereafter, the solvents were evaporated using a vacuum rotary evaporator. Sodium methylate (CH_3_ONa) was used to obtain the fatty acids’ methyl esters. Fatty acid methyl esters were analyzed by GC-FID (Clarus 500, PerkinElmer, Shelton, CT, USA) with a flame ionization detector and column EC™-WAX, 30 m × 0.25 mm ID, 0.25 μm film thickness. The atherogenic index (AI) and thrombogenic index (TI) were calculated using the equations of Ulbricht and Southgate [19].

For minor fatty acids present at trace levels, the relative analytical uncertainty is higher near the method quantification limit. Therefore, small numerical differences were interpreted cautiously and emphasized only when supported by statistical analysis.

### 2.4. Antioxidant Indicators and Lipid Peroxidation Assays

DM samples were analyzed for antioxidant indicators (enzyme activity), including superoxide dismutase (SOD) activity, catalase (CAT) activity, and glutathione (GSH) levels using methods of Sun et al. [20], Aebi [21], and Akerboom and Sies [22].

MDA concentration was measured by the method of Plaser et al. [23].

To determine the glutathione peroxidase (GPx-1), additional lipid peroxidation (LPO) was performed with ELISA kits (Abcam, Cambridge, UK) following the manufacturer’s instructions.

The spectrophotometric analysis of TAC, i.e., the total antioxidant activity assessment of the DM, was performed according to the methods of Brambilla et al. [24] and Bianchi et al. [25], after additional adaptation. Each DM sample was tested in triplicate, after dissolution in hypochlorous acid (HClO) and N, N-diethylparaphenylenediamine, and the absorbance was measured at 546 nm until a colored complex formed. TAC values are expressed in μmol.

### 2.5. Electron Paramagnetic Resonance (EPR)-Based Redox Marker Analysis

In heterogeneous systems, the EPR spin-trap method is used to detect short-lived radicals (R•) [26] and to assess molecular mobility and organization in complex matrices such as milk [27]. The measurements were performed with an X-Band, EMX micro-spectrometer (Bruker) with the following settings: center field 3505 G; sweep width 10–30 G; microwave power 12.70–12.83 mW; receiver gain 1 × 10^4^–1 × 10^6^; mod. amplitude 5.00 G; and an accumulation of 1–5 scans. Experiments were carried out in triplicate, and the EPR spectrum was immediately registered. Spectral processing was performed using Bruker, WIN-EPR version 2021 and SimFonia software version 2021.Rationale for marker selection: The selected panel was designed to capture complementary domains of milk redox-related properties. Classical enzymatic antioxidants (SOD, CAT, GPx-1) and non-enzymatic defenses (GSH, TAC) provide an overview of antioxidant capacity, while MDA/LPO reflect lipid peroxidation. In addition, EPR-based assays provide direct or functional readouts related to radical-scavenging capacity and redox buffering (DPPH, Asc•, ROS- and NO•-related signals, TEMPOL conversion, and thiol/protein oxidation assessed by 5-MSL). This combined approach supports a more integrated interpretation of lactation-stage effects on milk redox status.

(1) DPPH scavenging ability

The radical-scavenging ability of donkey milk (DM) was assessed using the DPPH (2,2-diphenyl-1-picrylhydrazyl) assay following the method of Shi et al. [28] and Qu et al. [29], with adaptations for EPR readout. Briefly, DPPH/ethanol solution was homogenized and incubated with DM for 5 min (final DPPH and sample concentrations as specified above). An aliquot of the reaction mixture was transferred into the Micro-221-EPR cavity at 23 °C, and DPPH-H/R signal evolution was recorded immediately. Results are presented as % DPPH scavenging activity.

(2) Ascorbate (Asc•) radicals

The method of Buettner and Jurkiewicz [30] was used to evaluate the ascorbate levels (Asc•) and their protection against oxidative toxicity. In brief, 200 mg DM was homogenized in cold dimethyl sulfoxide (DMSO) (10% *w*/*v*) and centrifuged at 4000× *g* for 10 min, at 4 °C. Supernatants were transferred into Eppendorf and immediately analyzed with the following settings: 3505 G centerfield, 6.42 mW microwave power, 5–10 G modulated amplitude, 1–5 scans. The spin-adducts formed between DMSO and generated Asc• radicals were recorded in real time.

(3) ROS production

One hundred μL of DM was homogenized with 900 μL of 50 mM PBN in DMSO using a sonicator (Sonopuls HD 2070, BANDELIN Electronic GmbH & Co. KG, Berlin, Germany) (one cycle, 1 min). After 5 min of incubation on ice, the suspension was centrifuged at 4000 rpm for 10 min at 4 °C and analyzed immediately. The real-time ROS production in the supernatants was estimated as described earlier [28], with some modifications [31,32].

(4) Nitric (NO•) radicals

Nitric oxide (NO•) was assessed by EPR using the spin trap carboxy-PTIO (CPTIO.K) following established methods [33,34]. Briefly, 50 μM CPTIO.K was prepared in 50 mM Tris buffer (pH 7.5) with DMSO (9:1), centrifuged at 4000× *g* for 10 min at 4 °C, and then mixed 1:1 with DM (100 μL + 100 μL) prior to EPR recording.

(5) Protein (albumin) oxidation analysis

Protein oxidation (albumin thiol-group modification) in DM was assessed ex vivo by EPR using spin-conjugation with 3-maleimido proxyl (5-MSL). DM was mixed with 20 mM 5-MSL dissolved in 900 μL dimethyl sulfoxide (DMSO). The mixture was centrifuged (1000 rpm; 15 min) at 4 °C. The protein/albumin conformational (-SH) changes were recorded in triplicate, in random units, by the method described earlier [35].

(6) Superoxide anion radicals (O_2_•−) conversion

Piperidine nitroxide, 4-Hydroxy-2,2,6,6-tetramethylpiperidine 1-Oxyl (TEMPOL) solution (50 µL, 2 mM) was added to the DM samples and stirred on a vortex for 5 s at 23 °C. The reaction mixture (nitroxide/DM sample) was incubated for 10 min. The samples were placed in the EPR cavity and scanned twice [36].

Chemicals and spin-trap reagents were of analytical grade and were purchased from Sigma-Aldrich (St. Louis, MO, USA).

### 2.6. Statistical Analysis

Assumptions of normality and homogeneity of data variance were assessed using the Shapiro–Wilk and Levene’s test, respectively. Significant differences across lactation stages were performed using one-way ANOVA with Tukey’s post hoc test (α = 0.05).

The EPR mathematical analysis of spectra was performed using Bruker, WIN-EPR version 2021 and SimFonia software version 2021.

To examine associations between days postpartum and milk parameters, regression analysis was performed. Three models were compared: a linear model (1), a quadratic model (2), and a cubic model (3). Interpretation focused primarily on the effect size (R^2^/adjusted R^2^) rather than *p*-values alone. All regression analyses were based on n = 40 independent cross-sectional observations.

The models were defined as follows:

Y = a + bx(1)

Y = a + b_1_ x + b_2_ x^2^ (2)

Y = a + b_1_ x + b_2_ x^2^+ b_3_ x^3^ (3)

Y represents the respective parameter studied (antioxidants, etc.), x is the independent factor (time postpartum), and a, b_1_, b_2_, and b_3_ are the model coefficients.

All analyses were performed at a significance level of α = 0.05 using IBM^®^ SPSS^®^ Statistics 26.0.

## 3. Results

### 3.1. Milk Fat Content and Fatty Acid Profile

#### Fatty Acid Composition

The average percentage of milk fat and the energy value of DM during lactation are presented in Table 1. Milk fat decreased by late lactation (0–30 days: 0.70%; 61–90 days: 0.55%), with a corresponding decline in energy value. The highest value of DM fat (0.70 ± 0.014%) was observed during the first month of the lactation period. Both milk fat content and the energy value of dry matter in the first two lactation periods differed significantly from those measured in the third period.

The fatty acid composition of donkey milk dry matter is presented in Table 2. Although the numerical values of most fatty acids tended to be higher in the third lactation period (61–90 days postpartum), these differences were not statistically significant for the majority of parameters (ns, *p* > 0.05). A significant change was observed only for caproic acid (C6:0), where the third period differed from the earlier stages according to Tukey’s post hoc test. Oleic acid (C18:1) increased from the first to the second month of lactation and then declined by approximately 2.5% in the third period, although this variation was not statistically significant. 

**Figure 1:**
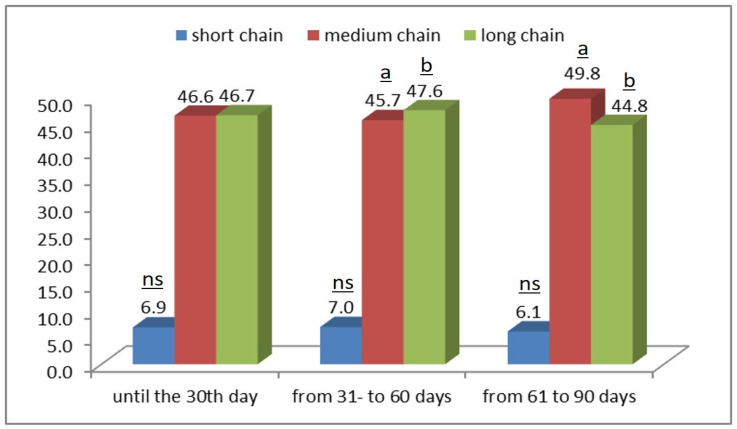
Changes in short-, medium-, and long-chain fatty acids during different lactation periods: up to 30 days, from 31 to 60 days, and from 61 to 90 days postpartum. Superscript letters indicate statistically significant differences (*p* < 0.05) between the second (31–60 days) and third lactation periods (61–90 days) for medium-chain fatty acids (a) and long-chain fatty acids (b); “ns” denotes non-significant differences. Post hoc analysis with Tukey’s test.

The content of short-chain fatty acids (C4–C11) (Figure 1) increased until the 60th day of lactation, after which it began to decrease, falling from 7% in the second month to 6.1% before the third month of lactation.

Medium-chain fatty acids (C12–C17) increased during lactation, rising from 46.6% in the first lactation period to 49.8% in the third month of lactation.

For long-chain fatty acids (above C18), the opposite trend was observed—they decrease by ~1.8% during lactation, with the highest amounts recorded between the 31st and 60th days of the lactation period. Statistically significant differences were observed for medium- and long-chain fatty acids between the second (31–60 days) and third (61–90 days) lactation periods (Tukey, *p* < 0.05), while changes in short-chain fatty acids were not significant.

**Figure 2:**
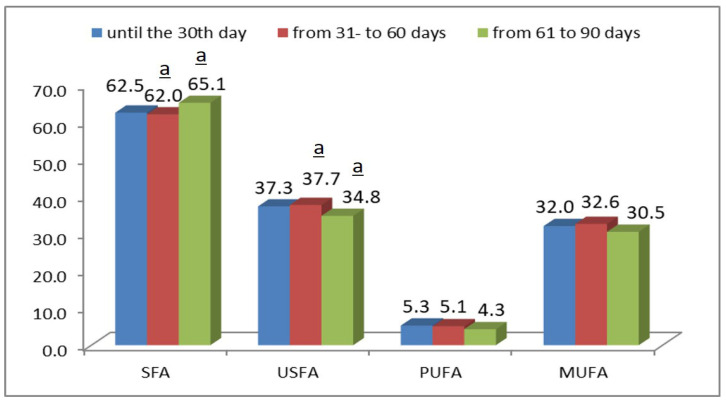
Fatty acid concentrations in DM from the Bulgarian local donkey breed during different lactation periods: up to 30 days, from 31 to 60 days, and from 61 to 90 days. Superscript “a” indicates statistically significant differences (*p* < 0.05) between the second (31–60 days) and third lactation periods (61–90 days) for the respective parameters. Post hoc analysis with Tukey’s test. SFA—saturated fatty acids; USFA—unsaturated fatty acids; PUFA—polyunsaturated fatty acids; MUFA—monounsaturated fatty acids.

The amount of saturated fatty acids in DM during the first month of lactation was 62.5%, after which it increased and reached 65.1% by the third month of lactation (Figure 2). Palmitic acid (C16:0) and myristic acid (C14:0) were the dominant saturated fatty acids and tended to increase by late lactation (C16:0: from 28.78% to 30.19%; C14:0: from 9.60% to 10.54%).

Oleic acid (C18:1) decreased by late lactation (from 29.36% to 27.97%); alpha-linolenic acid (C18:3) also decreased (from 1.61% to 1.01%).

In contrast to the increase in saturated fatty acids during lactation, a decrease was reported for unsaturated fatty acids. During lactation, their quantity decreased by approximately 3%, proportional to the rise in SFA. The most significant change was in MUFA content, which dropped from 32% in the first month of lactation to 30% by the third month. Statistically significant differences were observed for MUFA and PUFA concentrations between the second (31–60 days) and third (61–90 days) lactation periods (Tukey’s test, *p* < 0.05) while changes in SFA and USFA were not significant (Figure 2).

**Figure 3:**
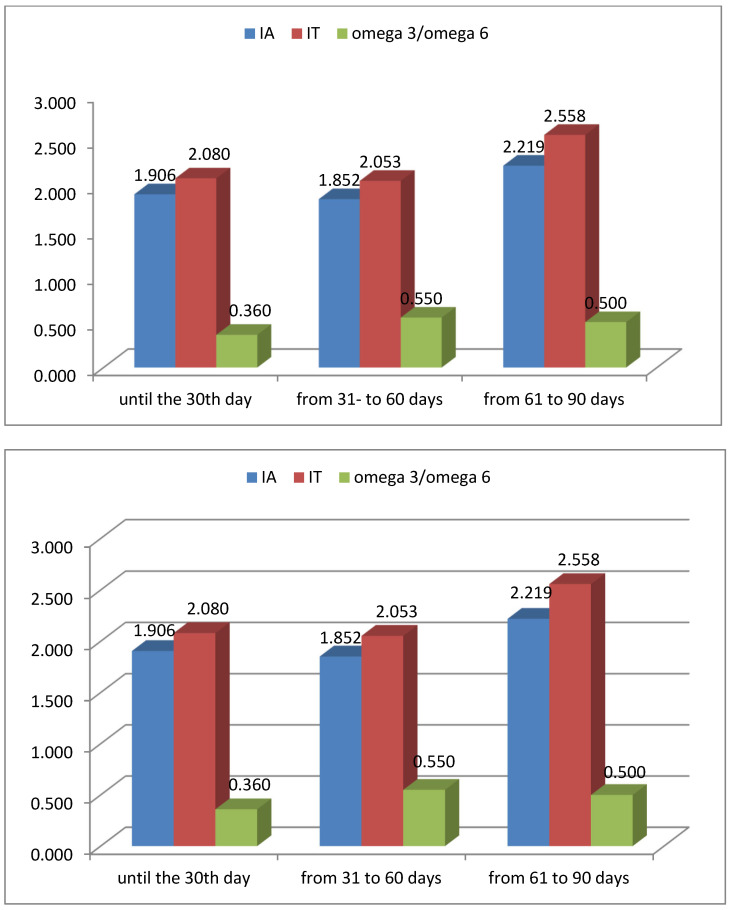
Health indices of DM during different lactation periods: up to 30 days; from 31 to 60 days; from 61 to 90 days. IA—index of atherogenicity; IT—index of thrombogenicity.

Consistent with these shifts, the atherogenic index increased from ~1.9 to ~2.2, and the thrombogenic index increased to ~2.5 (Figure 3). The smallest changes were observed in polyunsaturated fatty acids, which decreased by 1% during lactation.

The omega-3/omega-6 ratio in DM changed during the lactation period, increasing from 0.36 before the first month to 0.5 by the third month (Figure 3). In our study, two additional health indices were calculated—atherogenic and thrombogenic. The atherogenic index increased with advancing lactation—from 1.9 before the first month to 2.2 by the third month. The thrombogenic index, like the atherogenic index, increased with the advancement of lactation, reaching up to 2.5. Despite the increase in both indices over the course of lactation, they remained markedly lower than those reported for cow’s milk (IA—4.35 and IT—3.85, respectively) [37].

### 3.2. Antioxidant Activity of Donkey Milk

In the observed phase of the study, the enzyme SOD activity increased almost two-fold, from 2.66 ± 1.15 (until the 30th day) to 4.57 ± 0.99 (from 31 to 60 days) and then to a maximum of 5.05 ± 0.84 (from 61 to 90 days (Table 3)). This parameter, measured up to 30 days postpartum, differed significantly between 31–60 day and 61–90 day periods. The R^2^ coefficient (0.515) indicates a moderate influence of lactation time on the SOD activity, and moderate dismutation of the superoxide anion to H_2_O_2_, at >51%. No significant differences were observed in the CAT activities (2.77 ± 0.65; 2.56 ± 0.68; 3.14 ± 0.57) and GSH activities (22.18 ± 2.27; 20.59 ± 2.10; 20.51 ± 2.05) in the three studied time intervals, as shown by the low R^2^ coefficient (0.129) for CAT and R^2^ coefficient (0.116) for GSH concentrations. The lactation period had a slight effect on enzyme activity and a synchronous reduction in lipid peroxides and ROS production. In the last stage of the experiment, the GSH concentration decreased and reached 20.51 ± 2.05 U/gPr.

In addition, because DM contains MUFA and PUFA that are susceptible to peroxidation, MDA was used as an indicator of lipid peroxidation across lactation stages. The MDA value measured up to 30 days postpartum (5.86 ± 1.68) significantly differed from the values of this parameter measured up to 60 days (3.94 ± 0.95) and up to 90 days (4.07 ± 1.48) postpartum. No significant differences were noted for this parameter between the second and third groups. The R^2^ coefficient was not very high (0.300), indicating that direct lipid peroxidation (MDA) varies moderately with time, at 30%.

GPx-1 activity measured up to 30 days postpartum (232.21 ± 28.34 U/gPr) was significantly different from the values of this parameter measured up to 60 days (342.59 ± 46.52 U/gPr) and up to 90 days (391.39 ± 23.65 U/gPr) postpartum. Statistically significant differences in GPX-1 activity were noted between the second and third groups. GPx-1 activity was approximately 1.7-fold higher at 61–90 days than at 0–30 days postpartum (391.39 ± 23.65 U/gPr) (*p* < 0.05). The R^2^ coefficient (0.773) had high values, indicating that GPx-1 activity varies significantly with time in DM, i.e., GPx-1 activity increased with lactation stage, which is consistent with enhanced enzymatic antioxidant defense during later lactation.

Similar results were recorded with direct lipid radical (LPO) measurements. LPO measured 30 days (0.02 ± 0.03) and 60 days (0.04 ± 0.03) postpartum did not significantly differ from each other, but they significantly differed from those obtained ninety days postpartum (0.12 ± 0.15). The determination coefficient was low (0.198), indicating that only 19.8% of the variations in lipid radicals depend on the time postpartum.

TAC did not significantly vary during different lactation periods (at 30 and 60 days); it varied from a mean of 213.42 ± 3.92 µmol (until the 30th day) to a mean of 207.24 ± 8.45 µmol (from 30 to 61 days). Ninety days postpartum, TAC reached 199.75 ± 7.09 µmol, which significantly differed compared to the first two periods. The R^2^ value (0.386) suggests that lactation stage explains a modest proportion of TAC variability, indicating that non-enzymatic antioxidant capacity changes are present but not the dominant source of variation.

DPPH• scavenging activity remained approximately constant (~62%) across the three periods.

Asc• radicals measured up to 30 days and up to 60 days postpartum (Table 3), 0.005 ± 0.0006 a.u. and 0.005 ± 0.0015 a.u., were not statistically significant (*p* > 0.05). Ninety days postpartum, Asc• radicals increased to 0.075 ± 0.046 (*p* < 0.05), and statistically significant changes were found among the above two groups (*p* < 0.05). The coefficient of determination (R^2^) was 0.638, i.e., 63.8% of the variation in Asc• radicals can be explained by the influence of the lactation period.

ROS production in DM increased with increasing lactation period. The ROS and MDA content from the 30th day onward were significantly different from those at 31–60 days and 61–90 days. ROS products underwent a statistically significant, four-fold increase up to 30 days, up to 60 days, and up to 90 days postpartum (0.005 ± 0.0009; 0.006 ± 0.0003; 0.023 ± 0.004). Furthermore, ROS products showed statistically significant differences between 31 and 60 days and 61–90 days (*p* < 0.05). The high R^2^ coefficient (0.937) indicates that 93.7% of the ROS variations depend on the time postpartum.

In our study, the findings showed that NO• radicals measured up to 30 days of lactating animals reached 0.006 ± 0.0023 a.u., and increased three-fold and seven-fold 60 days (0.018 ± 0.003) and 90 days postpartum (0.023 ± 0.004). Furthermore, the NO• radical concentration up to 60 days and up to 90 days was significantly different. The high R^2^ coefficient (0.827) indicates that time significantly influences the variations in NO• radicals, in >82%. These data indicate lower NO•-related signals in early lactation, consistent with a lower nitrosative burden during the first 30 days postpartum.

In our study, using 5-MSL spin-trap, we assessed the free -SH groups conformation changes, antioxidant inhibition, and the state of antioxidant protection in DM. Through protein oxidation experiments in DM, it was found that the mean value of 5-MSL spin-trap measured 30 days postpartum was significantly higher, with a mean value of 38.57%, compared to 31–60 days and 61–90 days of lactation (*p* < 0.05). Statistically significant differences were noted between 60 and 90 days postpartum, with a mean of 21.65% vs. a mean of 4.17%, respectively. For this parameter, the R^2^ coefficient was high (0.897), indicating that 89.7% of free -SH group changes varied significantly for the tested periods. There was a maximum 5-MSL signal at 0–30 days postpartum, indicating the highest availability of free thiol (–SH) groups in early lactation. The decline at later stages is consistent with increased thiol oxidation during lactation.

Endogenous defense systems protect DNA and mitochondria from oxidative stress damage. In the present study, DM samples examined at 90 days postpartum had a significant increase in SOD, CAT, GPx-1 concentration, and a decrease in MDA levels, compared to the other groups. The range of O_2_•− radicals’ scavenging ability in DM measured until the 30th day (>96.1%) significantly differed from that measured up to 60 days (>86.5%) and up to 90 days (>86.5%) postpartum. No significant differences were observed in this parameter between 31 and 60 days and 61–90 days of lactation. The high R^2^ coefficient (0.922) indicates a strong dependence, >92% of the O_2_•− and almost complete conversion, and high TEMPOL conversion, indicating strong superoxide-related redox activity in donkey milk across the studied period. Our results indicated a higher radical-scavenging capacity of DM at 30 days postpartum that remained constant for the tested period.

TAC did not significantly vary during different lactation periods (at 30 and 60 days); it varied from a mean of 213.416 ± 3.9179 µmol (until the 30th day) to a mean of 207.239 ± 8.4379 µmol (from 30 to 61 days). Ninety days postpartum, TAC reached 199.748 ± 7.0926 µmol, significantly different compared to the 30th day and 31–60 days. The R^2^ value (0.386) suggests that lactation stage explains a modest proportion of TAC variability, indicating that non-enzymatic antioxidant capacity changes are present but not the dominant source of variation.

### 3.3. Regression Analyses

Table 4 presents the regression analyses showing how the fatty acid composition and antioxidant and redox-related properties of donkey milk change during the lactation period.

**Figure 4:**
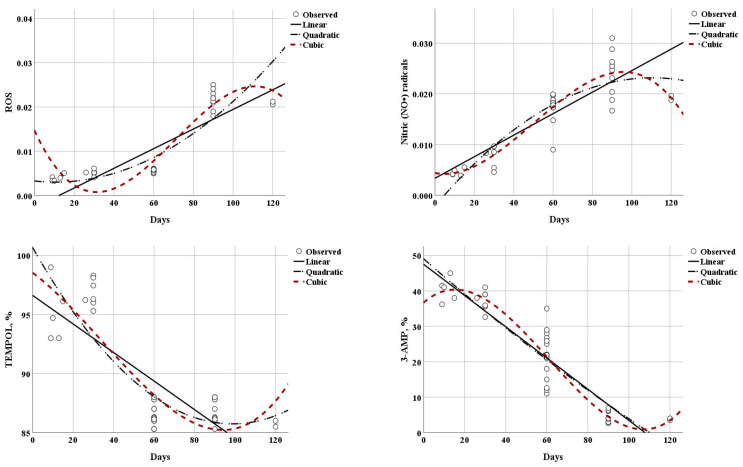
Estimation of the regression models’ curves showing the changes in fatty acid composition, and the antioxidant and redox-markers related to donkey milk, depending on the time postpartum.

Figure 4 illustrates fitted curves for selected regression models describing changes in milk composition and redox-related parameters over days postpartum. Statistically significant associations were obtained for most parameters (*p* < 0.05), whereas CAT models were not significant. To limit overfitting and to keep interpretation parsimonious, we primarily emphasize linear and quadratic fits and interpret the results based on effect size (R^2^/adjusted R^2^) and biological plausibility, rather than on *p*-values alone. Higher-order (cubic) fits were explored only as an exploratory comparison and are not used as the main basis for biological interpretation. The highest R^2^ coefficients were obtained for the parameters: –SH groups and protein (albumin) oxidation (5-MSL; R^2^ coefficient = 90.0%), NO• radicals (R^2^ coefficient = 85.9%), ROS production/ lipid peroxidation (R^2^ coefficient = 82.2%), and highest biological response to O_2_•− and HO• radicals (TEMPOL; R^2^ coefficient = 74.9%), meaning that the highest percentage of variations in these variables can be explained by the lactation period. The developed models for these parameters show the highest predictive efficiency, R^2^ coefficient = 75–90%, among the studied characteristics.

## 4. Discussion

### 4.1. Fat Content, Fatty Acid Profile, and Health-Related Indices

In this study, donkey milk showed the highest fat content in early lactation, followed by a gradual decline toward later lactation. Similar stage-dependent patterns have been reported in other donkey breeds and may reflect shifts in mammary lipid synthesis, nutrient partitioning, and energy balance across lactation [38,39,40].

Because donkey milk is naturally low in fat, its energy contribution is limited. When considered for infant or clinical nutrition, it should be evaluated within the total diet, and fortification or complementary feeding may be required to meet energy needs [41,42,43,44]. DM from the Bulgarian native breed is characterized by low fat content, which contributes to its lower energy value. A modest decline in fat content toward late lactation may reflect stage-dependent changes in mammary lipid synthesis and secretion as well as changes in the animals’ energy balance over lactation.

Oleic acid (C18:1), the most abundant of the monounsaturated fatty acids, is a major source in human nutrition in many countries [45]. Across the first 90 days postpartum, the fatty acid profile shifted toward higher total SFA and lower total MUFA, including a decline in oleic acid (C18:1) and alpha-linolenic acid (C18:3) toward late lactation. A high percentage of unsaturated fatty acids in human nutrition has a favorable effect on LDL and total cholesterol levels [46]. Differences among studies and breeds are expected, as feeding system, season, and management can influence fatty acid supply and mammary lipid metabolism [38,41,47,48,49].

The omega-6/omega-3 ratio and its stage-related changes are plausibly driven by dietary fatty acid intake; unlike ruminants, equines do not biohydrogenate dietary unsaturated fatty acids in the foregut, so the dietary signal may be more directly reflected in milk fat [50,51].

Health-related lipid indices (atherogenicity and thrombogenicity) increased with advancing lactation in parallel with the shift toward more saturated profiles. Although higher IA/IT values are generally considered less favorable for cardiometabolic risk assessment, these indices remain substantially lower than values typically reported for cow’s milk, supporting the view that donkey milk retains a comparatively favorable lipid profile overall [52,53,54].

Mechanistically, the increase in SFA (notably C14:0 and C16:0) together with the decline in MUFA/PUFA can explain the rise in IA and IT, because these indices weigh pro-atherogenic saturated fatty acids more strongly. The observed increase in SOD, CAT, and GPx-1 with advancing lactation may reflect an adaptive upregulation of enzymatic defenses in response to higher metabolic load and increased ROS/RNS generation.

### 4.2. Antioxidant Activity and Radical-Scavenging Potential

Oxidative and nitrosative processes can affect milk quality by influencing lipid stability, protein thiol status, and the overall antioxidant potential of the milk matrix. To capture complementary domains of redox-related properties, we combined enzymatic antioxidants, non-enzymatic indices, and EPR-derived markers across early, mid, and late lactation [55].

Overall, later lactation was characterized by higher ROS- and NO•-related signals alongside higher lipid peroxidation markers, indicating a greater oxidative challenge in milk at this stage. At the same time, EPR-based indicators of thiol status (5-MSL) suggested reduced availability of protein -SH groups toward late lactation, consistent with thiol oxidation and a reduced thiol-buffering capacity.

Enzymatic antioxidant defenses (SOD and GPx-1) increased with advancing lactation, whereas CAT and GSH changed little. This pattern is compatible with a compensatory upregulation of endogenous defense pathways in response to higher reactive species generation, rather than a simple improvement in overall redox stability. SOD converts O_2_•− to H_2_O_2_, and GPx-1 reduces peroxides using glutathione, limiting propagation of oxidative chain reactions [56,57].

In contrast, non-enzymatic readouts showed smaller or opposite shifts: DPPH• scavenging remained relatively stable, while TAC declined toward late lactation. These divergent trends are biologically plausible because TAC largely reflects low-molecular-weight antioxidant pools (e.g., vitamin-related reducing equivalents), which can be consumed or diluted over time, whereas enzyme activities reflect inducible defense capacity. Together, these measures suggest that early lactation has stronger non-enzymatic buffering, while later lactation relies more on enzymatic compensation. Changes in Asc•-related signals are compatible with lactation-stage differences in vitamin C-related reducing equivalents and/or dietary supply [58,59].

We observed an increase in enzymatic antioxidant activities (SOD and GPx-1) with advancing lactation, together with a decrease in TAC. These divergent trends are biologically plausible because TAC primarily reflects non-enzymatic antioxidant pools (e.g., vitamins, urate, low-molecular antioxidants), whereas enzyme activities reflect endogenous antioxidant defense capacity. As lactation progresses, metabolic demand and reactive species generation may rise, stimulating compensatory upregulation of enzymatic defenses, while non-enzymatic antioxidants can be consumed or diluted, resulting in lower TAC.

Because several fatty acids occur at trace levels, small numerical between-stage differences may fall within analytical variability; therefore, we primarily interpret differences supported by the statistical analysis and biologically meaningful effect sizes.

Limitations include the cross-sectional design of the cohort, the lack of inflammatory cytokine measurements, and the lack of clinical outcomes in animals or consumers. Future longitudinal studies integrating cytokines, iNOS-related endpoints, and standardized redox panels would better define redox-inflammatory relationships.

## 5. Conclusions

Donkey milk from the Bulgarian local donkey breed displayed clear lactation-stage variability in both lipid composition and redox-related properties. Monounsaturated fatty acids decreased from approximately 32% in early lactation to about 30% in late lactation, while IA increased from ~1.9 to ~2.2, and IT increased to ~2.5. Enzymatic antioxidant activities (SOD and GPx-1) increased with lactation, whereas total antioxidant capacity declined (213.4 to 199.7 µmol) and DPPH radical-scavenging activity remained stable. EPR-derived redox markers indicated stage-dependent changes, including higher ROS- and NO•-related signals at later lactation and a marked decrease in the thiol-related 5-MSL signal. From a management perspective, late lactation may benefit from targeted nutritional antioxidant support and monitoring of redox markers, while early lactation milk may be preferable when higher thiol-based redox buffering is desired for functional dairy applications. These findings support stage-specific interpretation of donkey milk nutritional quality and redox stability and motivate future longitudinal work linking milk biomarkers to animal health, management practices, and consumer-relevant outcomes.

## Figures and Tables

**Table 1:** Dynamics of milk fat (%) content and energy value (kcal/g) of DM during the lactation period: from 1 to 30 days; from 31 to 60 days; from 61 to 90 days.

Lactation Periods (Days)	Fat (%)	Energy Value (kcal/g)
until the 30th day	0.70 ± 0.014 ^a^	42.18 ^a^
from 31 to 60 days	0.69 ± 0.013 ^b^	42.33 ^b^
from 61 to 90 days	0.55 ± 0.016 ^ab^	39.51 ^ab^

Identical superscripts within columns indicate significant differences (*p* < 0.05) as follows: ^a^—between mean values of investigated parameters up to the 30th day and all other periods; ^b^—between mean values of investigated parameters from 31 to 60 days and from 61 to 90 days. Post hoc analysis with Tukey’s test.

**Table 2:** Dynamics of the fatty acid composition evaluated in DM at different lactation periods.

Fatty Acids	Until the 30th Day	From 31 to 60 Days	From 61 to 90 Days
	$\bar{x}\pm SD$	$\bar{x}\pm SD$	$\bar{x}\pm SD$
	Short-chain fatty acids		
Butyric acid (C4:0)	0.870 ± 0.051 ^ns^	0.890 ± 0.051 ^ns^	0.900 ± 0.065 ^ns^
Caproic acid (C6:0)	1.983 ± 0.109 ^ab^	1.900 ± 0.109 ^ab^	1.153 ± 0.123 ^a^
Caprylic acid (C8:0)	0.673 ± 0.065 ^ns^	0.693 ± 0.065 ^ns^	0.740 ± 0.057 ^ns^
Capric acid (C10:0)	3.432 ± 0.114 ^ns^	3.293 ± 0.114 ^ns^	3.093 ± 0.355 ^ns^
Undecanoic acid (C11:0)	0.254 ± 0.031 ^ns^	0.243 ± 0.031 ^ns^	0.235 ± 0.020 ^ns^
Medium-chain fatty acids			
Lauric acid (C12:0)	3.033 ± 0.220 ^ns^	2.797 ± 0.165 ^ns^	3.177 ± 0.251 ^ns^
Tridecanoic acid (C13:0)	0.160 ± 0.022 ^ns^	n.a.	0.173 ± 0.006 ^ns^
Myristic acid (C14:0)	9.603 ± 0.100 ^ns^	9.423 ± 0.451 ^ns^	10.543 ± 0.533 ^ns^
Myristoleic acid (C14:1)	0.300 ± 0.019 ^ns^	0.287 ± 0.026 ^ns^	0.450 ± 0.048 ^ns^
Tetradecadienoic acid (C14:2)	0.140 ± 0.012 ^ns^	0.140 ± 0.000 ^ns^	0.315 ± 0.014 ^ns^
Anteiso pentadecanoic acid (C15 iso)	0.293 ± 0.036 ^ns^	0.340 ± 0.038 ^ns^	0.585 ± 0.032 ^ns^
Pentadecanoic acid (C15:0)	0.827 ± 0.038 ^ns^	0.723 ± 0.068 ^ns^	1.003 ± 0.084 ^ns^
Palmitic acid (C16:0)	28.777 ± 0.456 ^ns^	28.527 ± 0.310 ^ns^	30.187 ± 0.560 ^ns^
Palmitoleic acid (C16:1)	2.123 ± 0.089 ^ns^	2.107 ± 0.089 ^ns^	1.727 ± 0.176 ^ns^
Hexadecadienoic acid (C16:2)	0.353 ± 0.068 ^ns^	0.387 ± 0.068 ^ns^	0.400 ± 0.017 ^ns^
Heptadecanoic acid (C17:0)	0.737 ± 0.040 ^ns^	0.677 ± 0.082 ^ns^	0.880 ± 0.106 ^ns^
Cis-10-heptadecanoic acid (C17:1)	0.223 ± 0.059 ^ns^	0.330 ± 0.000 ^ns^	0.313 ± 0.022 ^ns^
	Long-chain fatty acids		
Iso stearic acid (C18 iso)	2.960 ± 0.131 ^ns^	2.803 ± 0.192 ^ns^	3.163 ± 0.195 ^ns^
Stearic acid (C18:0)	9.290 ± 0.178 ^ns^	9.777 ± 0.375 ^ns^	9.573 ± 0.190 ^ns^
Oleic acid (C18:1)	29.363 ± 0.486 ^ns^	30.097 ± 0.360 ^ns^	27.973 ± 0.368 ^ns^
Linoleic acid (C18:2)	3.233 ± 0.211 ^ns^	3.000 ± 0.096 ^ns^	2.817 ± 0.236 ^ns^
Linolenic acid (C18:3)	1.607 ± 0.138 ^ns^	1.660 ± 0.148 ^ns^	1.010 ± 0.211 ^ns^
Conjugated linoleic acid (CLA)	0.200 ± 0.004 ^ns^	0.260 ± 0.030 ^ns^	0.300 ± 0.001 ^ns^

Identical superscripts within rows indicate significant differences (*p* < 0.05) as follows: ^a^—between mean values of investigated parameters up to the 30th day and all other periods; ^b^—between mean values of investigated parameters from 31 to 60 days and from 61 to 90 days; ^ns^—insignificant differences; post hoc analysis with Tukey’s test; SD—standard deviation, n.a.—not analyzed.

**Table 3:** Results of the variance analysis of the studied ROS/ RNS parameters, lipid peroxidation, and enzymatic antioxidants, depending on the three investigated periods (one-way ANOVA with post hoc Tukey’s test) postpartum.

Variable	Until the 30th Day (*n* = 12)	From 31 to 60 Days (*n* = 16)	From 61 to 90 Days (*n* = 12)	R^2^
	$\bar{x}\pm SD$			
SOD (U/gPr)	2.66 ± 1.15 ^a^	4.57 ± 0.99 ^a^	5.05 ± 0.84 ^a^	0.515
CAT (U/gPr)	2.77 ± 0.65	2.56 ± 0.68	3.14 ± 0.57	0.129
GSH (U/gPr)	22.18 ± 2.27	20.59 ± 2.10	20.51 ± 2.05	0.116
MDA (µmol/mL)	5.86 ± 1.68 ^a^	3.94 ± 0.95 ^a^	4.07 ± 1.48 ^a^	0.300
GPx-1 (U/gPr)	232.21 ± 28.34 ^a^	342.59 ± 46.52 ^ab^	391.39 ± 23.65 ^ab^	0.773
LPO (%)	0.02 ± 0.03 ^a^	0.04 ± 0.03 ^b^	0.12 ± 0.15 ^ab^	0.198
TAC (µmol)	213.42 ± 3.92 ^a^	207.24 ± 8.45 ^b^	199.75 ± 7.09 ^ab^	0.386
DPPH• (arb.units,%)	61.75 ± 4.14	61.44 ± 3.81	61.75 ± 4.14	0.002
Asc• (arb.units,%)	0.005 ± 0.0006 ^a^	0.005 ± 0.0015 ^b^	0.075 ± 0.045 ^ab^	0.638
ROS (arb.units,%)	0.005 ± 0.0009 ^a^	0.006 ± 0.0003 ^ab^	0.023 ± 0.004 ^ab^	0.937
NO• (arb.units,%)	0.006 ± 0.0023 ^a^	0.018 ± 0.003 ^ab^	0.023 ± 0.004 ^ab^	0.827
5-MSL (arb.units,%)	38.57 ± 3.30 ^a^	21.65 ± 6.70 ^ab^	4.17 ± 1.43 ^ab^	0.897
TEMPOL (arb.units,%)	96.13 ± 1.93 ^a^	86.59 ± 0.90 ^a^	86.58 ± 0.99 ^a^	0.922

Identical superscripts within rows indicate significant differences (*p* < 0.05) as follows: ^a^—between mean values of investigated parameters up to 30th day and all other periods; ^b^—between mean values of investigated parameters from 31 to 60 days and from 61 to 90 days; post hoc analysis with Tukey’s test; SD—standard deviation; R^2^—coefficient of determination; n—number of the observations.

**Table 4:** Model summary and parameter estimation showing the changes in the antioxidant and redox-related properties of donkey milk, depending on the time postpartum, n = 40 (cross-sectional observations).

Parameter Estimates	R^2^	F	Sig.	Equations
Ascorbic (Asc•) radicals				
Linear	0.462	32.670	0.001	AR=−0.028+0.0009x
Quadratic	0.625	30.861	*p* < 0.001	$AR=0.015-0.001x+1.644{\;\times \;10}^{-5}x^{2}$
Cubic	0.642	21.551	*p* < 0.001	$AR=0.036-0.003x+4.968{\;\times \;10}^{-5}x^{2}-1.755x^{3}$
ROS				
Linear	0.631	64.868	*p* < 0.001	ROS=−0.003+0.0002x
Quadratic	0.705	44.192	*p* < 0.001	$ROS=0.003-4.786{\;\times \;10}^{-5}x+2.276{\;\times \;10}^{-6}x^{2}$
Cubic	0.822	55.468	*p* < 0.001	$ROS=0.015-0.001x+2.01{\;\times \;10}^{-5}x^{2}-9.41{\;\times \;10}^{-8}x^{3}$
LPO				
Linear	0.198	9.353	0.004	LPO=−0.021+0.0014x
Quadratic	0.240	5.852	0.006	$LPO=0.030-0.0009x+1.920{\;\times \;10}^{-5}x^{2}$
Cubic	0.241	3.801	0.018	$LPO=0.024-0.001x+1.0{\;\times \;10}^{-5}x^{2}+4.86{\;\times \;10}^{-8}x^{3}$
Nitric (NO•) radicals				
Linear	0.732	103.878	0.032	NR=0.003+0.0002x
Quadratic	0.811	79.157	0.011	$NR=-0.002-0.0005x-2.077{\;\times \;10}^{-6}x^{2}$
Cubic	0.859	73.246	0.050	$NR=0.01-7.6{\;\times \;10}^{-5}x+8.13{\;\times \;10}^{-6}x^{2}-5.4{\;\times \;10}^{-8}x^{3}$
DPPH				
Linear	0.010	0.375	0.045	DPPH=82.390+0.0002x
Quadratic	0.011	0.201	0.049	$DPPH=62.068-0.002x-0.0001x^{2}$
Cubic	0.121	1.649	0.019	$DPPH=67.26-0.42x+0.008x^{2}-4.28{\;\times \;10}^{-5}x^{3}$
TEMPOL, %				
Linear	0.617	61.173	*p* < 0.001	T=96.620−0.121x
Quadratic	0.728	49.479	*p* < 0.001	$T=100.676-0.303x-0.0016x^{2}$
Cubic	0.749	34.398	*p* < 0.001	$T=98.54-0.13x-0.002x^{2}-1.76{\;\times \;10}^{-5}x^{3}$
5-MSL, %				
Linear	0.851	217.202	*p* < 0.001	3−AMP=47.515−0.441x
Quadratic	0.853	107.198	*p* < 0.001	$3-AMP=49.082-0.511x-0.0006x^{2}$
Cubic	0.900	108.079	*p* < 0.001	$3-AMP=36.67+0.51x-0.02x^{2}+0.0001x^{3}$
MDA				
Linear	0.205	9.786	0.003	MDA=5.975−0.024x
Quadratic	0.220	5.514	0.008	$MDA=49.082-0.511x-0.0006x^{2}$
Cubic	0.231	3.611	0.022	$MDA=36.67+0.504x-0.019x^{2}+0.0001x^{3}$
SOD				
Linear	0.479	34.976	*p* < 0.001	SOD=2.220+0.033x
Quadratic	0.536	22.292	*p* < 0.001	$SOD=1.258+0.076x-0.0004x^{2}$
Cubic	0.548	14.520	*p* < 0.001	$SOD=1.08+0.09x-0.0006x^{2}+1.49{{\;\times \;10}^{-6}x}^{3}$
CAT				
Linear	0.049	1.969	0.169	CAT=2.50+0.005x
Quadratic	0.135	2.892	0.068	$CAT=3.022-0.018x-0.0002x^{2}$
Cubic	0.139	1.930	0.142	$CAT=3.18-0.03x+0.0004x^{2}-1.28{{\;\times \;10}^{-6}x}^{3}$
GSH				
Linear	0.104	4.410	0.042	GPX=22.46−0.02x
Quadratic	0.177	3.969	0.027	$GPX=24.038-0.094x-0.0006x^{2}$
Cubic	0.182	2.662	0.043	$GPX=24.65-0.15x+0.002x^{2}-5.118{{\;\times \;10}^{-6}x}^{3}$
GPX-1				
Linear	0.692	85.519	*p* < 0.001	GPX−1=203.58+2.04x
Quadratic	0.716	46.684	*p* < 0.001	$GPX-1=173.65+3.38x-0.011x^{2}$
Cubic	0.745	35.120	*p* < 0.001	$GPX-1=223.68-0.71x+0.07x^{2}-0.0004x^{3}$
TAC				
Linear	0.392	24.513	*p* < 0.001	TAC=217.57−0.18x
Quadratic	0.393	11.981	*p* < 0.001	$TAC=216.87-0.15x-0.0003x^{2}$
Cubic	0.397	7.911	*p* < 0.001	$TAC=214.61-0.03x-0.004x^{2}+1.86{\;\times \;10}^{-5}x^{3}$

Level of significance: *p* < 0.05. Model interpretation is based primarily on effect size (R^2^/adjusted R^2^) and model parsimony.

## Data Availability

The original contributions presented in this study are included in the article. Further inquiries can be directed to the corresponding author.
